# Transrectal Endoscopic Ultrasound‐Guided Drainage of a Deep Pelvic Abscess in a Postoperative Pediatric Patient: A Case Report

**DOI:** 10.1002/ccr3.71012

**Published:** 2025-09-29

**Authors:** Kareem Ibraheem, Mera Badareen, Amal M. Shawabka, Abdalrahman N. Herbawi, Maryam S. Mahareeq, Osama Hroub, Badawi Eltamimi

**Affiliations:** ^1^ Faculty of Medicine Palestine Polytechnic University Hebron Palestine; ^2^ Palestinian Clinical Research Center Bethlehem Palestine; ^3^ Gastroenterology Department Al Ahli Hospital Hebron Palestine

**Keywords:** appendicitis, deep pelvic abscess, endoscopic ultrasound, pelvic abscess, transrectal drainage

## Abstract

Transrectal Endoscopic Ultrasound‐Guided Pelvic Abscess Drainage (EUS‐PAD) is a minimally invasive technique for treating pelvic abscesses, especially those that are difficult to reach. It offers several advantages: reduced infection risk, shorter hospital stays, lower costs, less radiation exposure, and a bridge to safer, elective surgery.

## 1 Introduction

One of the most prevalent acute abdominal ailments is acute appendicitis. Postoperative abscess formation in the retroperitoneal area or the peritoneal cavity is one of its complications [1]. Acute surgical abdomen in children is frequently caused by appendicitis, which often manifests after a perforation. Percutaneous drainage of intra‐abdominal abscesses can be achieved through transabdominal, transgluteal, or transrectal approaches if the abscess is located deep within the pelvis [2].

Percutaneous drainage is not usually effective in treating pelvic abscesses. Radiologists are often unfamiliar with transrectal drainage of Douglas abscesses, which has been performed blindly by surgeons for many years. During the procedure, general anesthesia was used. In every case, the drainage catheter was well tolerated, and the post‐drainage hospital stay was brief. This approach can be applied regardless of the patient's age or gender if medical care fails. It utilizes drainage catheters and ultrasound transducers, which are commonly found in radiology departments [3].

## 2 Case Presentation

### 2.1 Case History and Examination

An 11‐year‐old female presented to the emergency department with severe generalized abdominal pain lasting for two weeks. The pain was dull, sudden in onset, and constant in nature, and it was associated with persistent fever of 38.9°C and vomiting. The pain coincided with her menstrual flow, and on examination, her abdomen exhibited rigidity and guarding.

### 2.2 Differential Diagnosis, Investigations and Treatment

**FIGURE 1:**
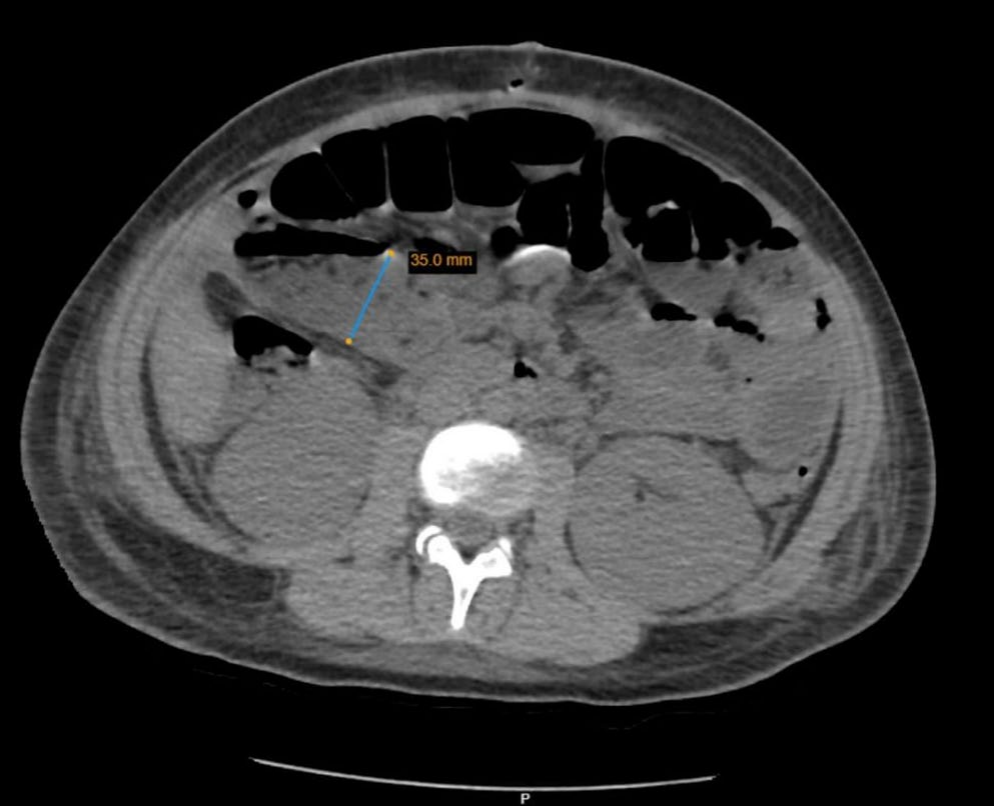
Abdominal CT scan of focal defect in the enhancing appendiceal wall, with extraluminal gas.

The initial differential diagnosis included appendicitis, gynecological conditions (such as ovarian torsion), and gastrointestinal infections. A complete blood count (CBC) revealed a white blood cell (WBC) count of 40,000 WBCs per microliter. An abdominal CT scan was performed and confirmed a perforated appendix (Figure 1).

The patient underwent a laparoscopic appendectomy, during which a significant amount of pus was found in the abdominal cavity. A postoperative drain was placed due to continued pus output. She was treated with analgesia and ceftriaxone antibiotics. Once the drain output ceased, the drain was removed. However, the patient's fever persisted at 39°C, necessitating a switch to vancomycin 500 mg every 6 h, with subsequent dose adjustments based on therapeutic drug monitoring to target trough levels of 10–20 mg/L and piperacillin/tazobactam 4.5 g every 8 h.

**FIGURE 2:**
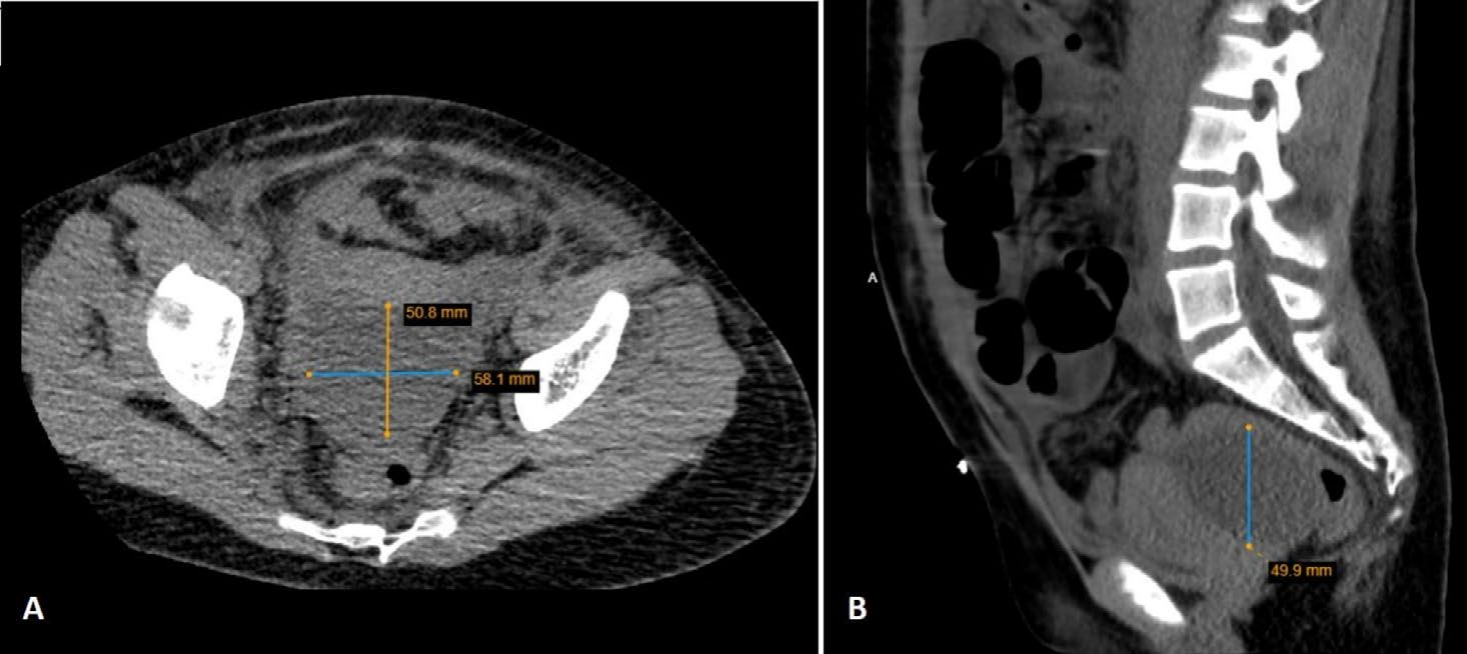
CT imaging of abscess formation in rectouterine excavation in transverse and sagittal plane (5 × 6 × 5.5 cm).

Four days post‐surgery, the patient experienced a recurrence of abdominal pain and fever, along with minimal generalized non‐pitting edema to the lower legs and increased abdominal distension. Pus also began discharging from the laparoscopic wound. The patient's initial hemoglobin was 12.3 g/dL and his creatinine was 0.9 mg/dL. Two days later, his hemoglobin was 11.1 g/dL and his creatinine was 1.3 mg/dL. An urgent abdominal computed tomography (CT) scan revealed an abscess in the Douglas pouch, measuring 5 × 6 × 5.5 cm (Figure 2).

Due to the patient's inability to tolerate further surgery and the challenging location of the abscess in the pouch of Douglas, which was not safely accessible via percutaneous drainage, a decision was made, in consultation with a gastroenterologist, to attempt drainage using a transrectal approach with endoscopic ultrasound (EUS) guidance.

**FIGURE 3:**
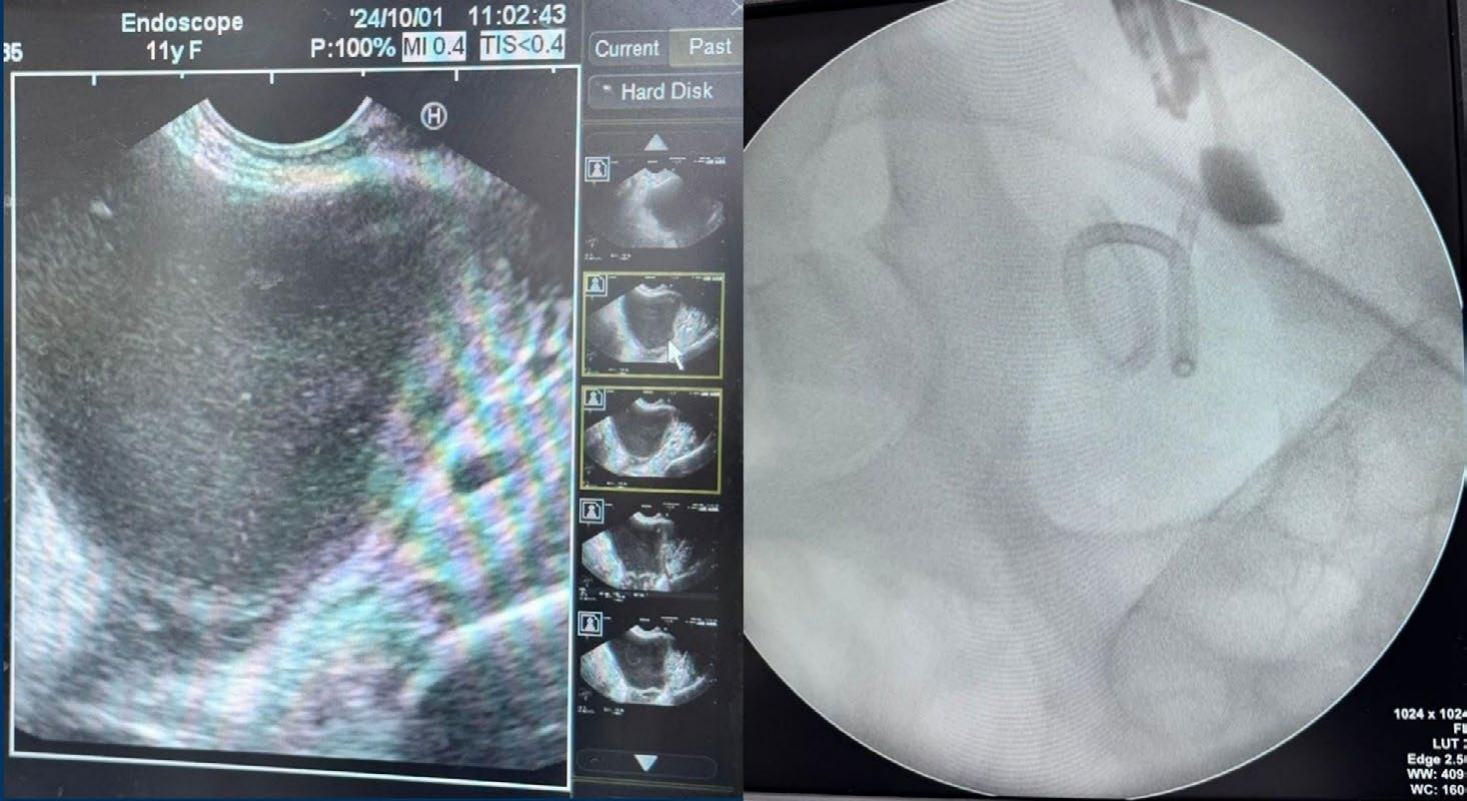
Ultrasound/Fluoroscopy imaging of collection in pouch of Douglas.

The procedure was performed under general anesthesia to ensure the patient's comfort and safety. Using an EUS scope, which provides high‐resolution ultrasound images, the abscess was visualized through the rectal wall. This imaging allowed for precise targeting of the collection. Under continuous EUS and fluoroscopic guidance (Figure 3), to prevent injury to adjacent organs, a needle was carefully advanced into the abscess cavity. Once its position was confirmed, a guidewire was placed, followed by dilation of the tract. A pigtail catheter was then inserted to allow continuous drainage of the abscess. The procedure was completed minimally invasively, avoiding the need for open surgery and reducing the risks associated with more aggressive interventions.

## 3 Conclusion and Results (Outcome and Follow‐Up)

The patient showed significant improvement one day after the procedure; her pelvic pain and fever subsided, and she was discharged with a 14‐day course of oral antibiotics. She was reevaluated in the outpatient clinic 10 days after discharge, where the sutures and drains were removed. Repeat ultrasound confirmed complete resolution of the abscess, and inflammatory markers (CRP and WBC count) had normalized. No rectal complications, such as bleeding, perforation, or fistula formation, were observed at this time; the incision was healing well, and there were no signs of infection. She was seen again 2 weeks later in the clinic, and no postoperative complications were noted. By this time, the patient had resumed normal daily activities without limitations.

## 4 Discussion

Acute appendicitis is the most frequent cause of abdominal surgery in children. There are many complications of appendicitis, including phlegmonous appendicitis, gangrenous non‐perforated appendicitis, and perforation [4]. The incidence of perforated appendicitis in children is significantly higher than in adults. This is due to their age, size, access to care, and other factors, with up to 50% of patients having an appendix perforation at the first visit [5]. It is widely known that abscess formation is a common complication following appendectomy, particularly in cases involving perforation [6]. Most complications are infections, typically involving wound infections, pelvic abscesses, or intra‐abdominal abscesses [7].

There are several studies recommending prophylactic measures to prevent intra‐abdominal or pelvic abscess formation after appendectomy, including the intraperitoneal administration of cefazolin at the end of laparoscopic appendectomy, which has been shown to reduce abscess occurrence [8]. However, other research indicates that postoperative antibiotics may not play a significant role in preventing abscess formation [9]. Additionally, in cases of perforated appendicitis undergoing laparoscopic appendectomy, the placement of an intraperitoneal drain does not appear to provide added benefit and may actually prolong hospital stays [10].

A pelvic abscess is a serious infection that typically develops after surgery or as a complication of medical conditions like pelvic inflammatory disease, appendicitis, or diverticulitis. Symptoms include fever, pelvic pain, and discharge. Early diagnosis using imaging and prompt treatment is critical. Treatment modalities include broad‐spectrum antibiotics as the first‐line option, with conservative management for stable patients with abscesses larger than 8 cm. Surgical drainage via laparotomy or laparoscopy is required for larger abscesses or those unresponsive to antibiotics. Transrectal endoscopic ultrasound (EUS)‐guided drainage is also effective for challenging cases. Treatment choice depends on abscess size, patient stability, and response to initial therapy [11].

Transrectal EUS is a procedure performed to drain pelvic abscesses, especially after surgery or abdominal infection [2, 12]. It involves positioning the patient in the left lateral decubitus position and inserting an ultrasound probe transrectally to visualize the abscess. Under real‐time imaging, a needle is advanced into the abscess cavity for aspiration, and a catheter is placed over a guidewire for continuous drainage. The catheter remains in place until the abscess resolves, monitored by follow‐up imaging. This minimally invasive technique is highly successful, with low complication rates, offering an effective approach for managing pelvic abscesses [12].

Although it is not the traditional method of treating pelvic abscesses, there are studies that showed it was effective in resolving abscesses, particularly in postoperative patients, with a low rate of complications and rapid recovery [2]. Also, it can be used in pelvic abscesses that cannot be safely drained via a percutaneous transabdominal or transvaginal route [13]. As in our case in which a CT scan identified a deep pelvic abscess in the Douglas pouch and showed that its location deep within the pelvis made percutaneous drainage challenging, risky, and can lead to injury to surrounding organs like the bladder, rectum, or uterus if not performed with extreme precision. Given the patient's clinical deterioration, the limitations of conventional surgical and percutaneous approaches, and depending on the previous theory which showed the high clinical success rate (92%) of the transrectal EUS in the drainage of deep pelvic abscesses, it was chosen as a less invasive and more targeted option [14].

There are similar cases where the percutaneous drainage wasn't feasible for 7 pediatric cases in which transrectal EUS was done and succeeded without any complications, leading to a short hospital stay [3].

Additionally, in pediatric patients, there was a retrospective series study that examined the safety and efficacy of transrectal EUS‐guided drainage for deep pelvic abscesses (DPAs) in children, typically caused by acute appendicitis. Six patients (mean age 12 years) were included, with DPAs treated using a lumen‐apposing metal stent for drainage under deep sedation. The procedure was technically successful in five out of six patients, who all recovered without recurrence or complications, revealing the method as a potentially safe and effective alternative to traditional percutaneous or surgical drainage methods when these are not feasible [15].

There are similar cases in which the patients have deep pelvic abscesses due to many surgical conditions, with their data summarized in (Table 1) compared to our case:

**TABLE 1:** Comparison of endoscopic ultrasound‐guided drainage (EUS‐PAD) in pelvic abscess management: Case studies and outcomes.

Case	First case	Second case	Third case	Our case
Reference	Goerl et al. (2023) [16]	Goerl et al. (2023) [16]	Khalid and Faisal (2021) [17]	—
Diagnosis	Retrovesical abscess due to sigmoid diverticulitis (Hinchey II)	Retrovesical abscess due to sigmoid diverticulitis (Hinchey II)	Perianal and perirectal abscesses in Crohn's disease	Abscess in Douglas pouch due to perforated appendicitis
Main symptom	Lower abdominal pain, chills, fever, tenderness, and acute kidney failure	Left‐sided lower abdominal pain, defensive tension, and elevated infection markers	Perirectal pain, swelling, fever, and fatigue	Severe generalized abdominal pain, fever, vomiting, and abdominal rigidity
Outcome	EUS‐guided drainage successfully reduced abscess size and improved renal function. The patient underwent elective laparoscopic resection three weeks later without complications. Discharged on the 10th postoperative day	EUS‐guided drainage led to rapid improvement in symptoms and inflammatory markers. Elective laparoscopic resection was performed eight weeks later without complications. Discharged on the 7th postoperative day	EUS‐guided drainage resolved the abscesses, and the patient improved significantly. Stent dislodged spontaneously, eliminating the need for endoscopic removal. No recurrence reported at follow‐up	EUS‐guided drainage successfully resolved the abscess. Patient improved within a day, discharged with oral antibiotics. Follow‐up confirmed complete resolution of the abscess and normalization of inflammatory markers. No complications observed

Although EUS alone can visualize the abscess [18], fluoroscopy adds an extra layer of safety and confirmation, particularly when the abscess is deeply located or has thick contents [19]. This is the cause that we used fluoroscopy in our case as the abscess was in the pouch of Douglas, in which the needle and catheter can injure the adjacent structures, including the rectum, bladder, uterus, posterior vaginal wall, iliac vessels, and portions of the bowel such as the sigmoid colon or small intestine [20], so despite the requirement for anesthesia and the associated drawbacks of increased radiation exposure from fluoroscopy [21], the specific location of the abscess necessitates its use during transrectal EUS drainage.

While endoscopic drainage of pelvic abscesses hasn't yet been included in major medical guidelines, the current supporting evidence consists primarily of a limited number of retrospective case studies and case reports [22, 23]. To effectively evaluate and compare treatment outcomes, as well as to reassess the appropriate first‐line interventions for pelvic abscesses, particularly in patients unsuitable for conventional methods, such as those who cannot tolerate surgery or those with deep abscesses where alternative access routes may pose safety risks, there is a pressing need for additional case reports and larger prospective studies.

### 4.1 Limitations

This report details a single case, which inherently limits the generalizability of its findings. While our patient had a favorable outcome, the efficacy and safety of transrectal EUS‐guided drainage in the broader pediatric population can't be definitively established from this case alone. We also didn't have long‐term follow‐up beyond six months, so we can't fully assess any potential late complications or recurrence. It's also important to consider potential biases in how we selected this patient. The decision to go with EUS‐guided drainage instead of traditional surgery was heavily influenced by the patient's instability and inability to undergo another operation. This could mean there's a selection bias, as more stable patients or those with easier‐to‐access abscesses might benefit more from other approaches. Lastly, even though EUS‐guided drainage generally involves less radiation than CT‐guided procedures, using fluoroscopy in a child still means some radiation exposure. This is always a concern in pediatric cases and should be minimized whenever possible.

## Author Contributions

**Kareem Ibraheem:** conceptualization, data curation, formal analysis, methodology, validation, writing – original draft, writing – review and editing. **Mera Badareen:** conceptualization, formal analysis, writing – original draft, writing – review and editing. **Amal M. Shawabka:** conceptualization, formal analysis, writing – original draft, writing – review and editing. **Abdalrahman N. Herbawi:** conceptualization, methodology, validation, writing – original draft. **Maryam S. Mahareeq:** formal analysis, investigation, resources, writing – review and editing. **Osama Hroub:** conceptualization, methodology, project administration, writing – original draft. **Badawi Eltamimi:** conceptualization, data curation, methodology, supervision, validation, writing – review and editing.

## Consent

Written informed consent was obtained from the patient's family for the publication of this case report.

## Conflicts of Interest

The authors declare no conflicts of interest.

## Data Availability

The data used to support the findings of this study are included in the article.
